# Ierne Speaks out and Strikes Home

**Published:** 1919-06-21

**Authors:** 


### THE COLLEGE AND ITS ENEMIES.

### Ierne Speaks Out and Strikes Home.

Terminology and toxicology are the weapons which the enemies of the College of Nursing use in conducting their campaign of frenzy. In the vernacular, they are spitting poison, and the College might be able to afford to continue its dignified attitude of silence and aloofness if the propagandists confined their operations to the domestic circle. But the virus is being distributed broadcast, and there is evidence that it is also being absorbed. The experienced nurse who knows the value of the work which the College has achieved, and likewise the barren record of its critics, is able to appreciate both at their true worth. Not so the populace. Not so even men who sit in the House of Lords. *Vide* Lord Ampthill, who suggests that if nurses are not granted State Registration, they will be forced to form a trade union. With due respect, and without intent to offend, his Lordship's is an ignorant remark. State Registration bears no relation to Trade Unionism. It would be as logical to say that if nurses do not powder their faces they will be forced to form a trade union. Trade unions confine their efforts to the obtaining of shorter hours of toil and higher remuneration, and the most elementary student of economics must realise that the passage of a Registration Bill through Parliament cannot affect either, immediately or ultimately. School teachers have had a State Register for years, but there was recently a teachers' strike in Wales. I am doubtful if Lord Ampthill can have given this matter more than a passing thought. He is probably the victim of poisonous propaganda.

### A Man of Science Joins the Mob.

It is a matter as painful as it is surprising to find a man of science appealing to the passions of the mob. At the crucial hour the whole world is aflame. The agents of Bolshevism, Socialism, and Anarchy are busily engaged in setting employers and employees at each other's throats. "Red" armies and "White" are engaged in bloody conflict, and "Red" stands not for justice or high ideals, but for licence and greed. To this surging sea of fire Mr. Paterson appeals in the columns of the *Times*. The College Bill, he affirms, is the Employers' Bill. That of the Central Committee is the Bill of the rank and file.

### Think this Matter Out!

I want the reader to think this matter right out. It is no trifling affair, but one pregnant with vital issues. In the first place Mr. Paterson's statement is false. But leaving aside its falsity, the appeal is unworthy. It is a deliberate and insidious attempt to introduce discord and strife within the walls of the country's noblest institutions—to cause the nurse to regard the Hospital Committee and the hospital matron as her natural enemies. Think it out, reader. Do not take my word for it, or the word of anybody. The facts can be verified and analysed, and truth can be separated from falsehood by process as unerring and scientific as alloy can be separated from gold. The person who fails to take the trouble to discern is, obviously, the willing victim of the propagandists of clap-trap.

### The Process.

Begin at the source. What man, or class of man, founds, establishes, and pays for a voluntary hospital? What man, or class of man, benefits from its ministrations? A voluntary hospital, in my estimation, brings together in manner perfectly unique, and unites with Christ-like bond the very rich and the very poor. That some of the women who labour there have been underpaid and overworked is undeniably true. But it is equally true that the sordid motive is not only absent, but impossible. No "employer" in the hospital world profits a single farthing from the labours of the nurse. If a draper or a builder sweats his workers it is to add to his personal income. The nurse is sweated in the interest of the sick-poor. Hence the enormity of the offence of endeavouring to alienate the nurse from her matron and her committee, and to jeopardise thereby the very existence of the only institutions in the country where the poor can come for skilful treatment in the stress of sickness. One would have thought that in the world of commerce, not to mention the hospital world, every man who has at heart the nation's prosperity should find it a pleasurable duty to promote harmony rather than discord. It is not by gathering in hostile camps, but by comradeship and co-operation that employer and employee will bring benefit to themselves and to the nation. This surgeon's appeal is for a divided camp. And, speaking for myself, I should be the last to cast a stone if he or any of the Central Committee of which he is the mouthpiece had ever at any time successfully championed the nurse in her claim for economic betterment. She is, speaking generally, a simple woman devoted to her duty, often times weary to death, while some are underpaid, and with little outlook. The Central Committee boasts a thirty years' existence. I invite any whom these remarks concern **to point to any public action ever taken by the Central Committee to redress the economic wrongs of the nurse.** For every ill their universal panacea is a register of names at Westminster. I sometimes wonder if they ever thought of sending their prescription to Messrs. Lenin and Trotski!

### An Offspring of the Red Cross

The College of Nursing is a war product. It is the offspring of the Red Cross. Florence Nightingale and the trained nurse are war products, the offspring also of the Red Cross. The College has a policy, but much more than a policy. It has a mission, and its mission, in a word, is the unification and emancipation of British nurses. In the dead days of peace the nurse's value to the nation was unappreciated and forgotten. But when England found herself in the claws of the Beast her men took up arms and her women took up nursing. Side by side they fought, each in their respective capacities, and, heaven be thanked, they won. I ask for no better testimony to the value of the College's mission than that of Mr. Paterson. He described it as a State Registration Society, a benevolent institution, and a university. In recent weeks it has proved itself a pioneer in the field of economics. The report on nurses' salaries published by the College is a document worthy of a Royal Commission, and its effects will be far-reaching.

### "Nurses, Up and at 'Em!"

I reckon amongst my valued acquaintances a great St. Bernard dog, an animal as beautiful as he is large. Again and again I have admired him following his master in his morning ride, and I have seen mongrels of considerable proportions yapping at his heels. Never once have I seen him turn his head to notice them. I always feel like saluting that dog as he strides along. The College of Nursing is the object of organised attack. The enemy is spitting poison, and I suggest the time has come for an organised truth propaganda. The "rank and file" are being inspired to revolt against their "employers" at a time when the relations between employer and employed are strained to the uttermost in both hemispheres. The College stands for regeneration, reconstruction, enlightenment. Its enemies are out to destroy. Let the rank and file speak. There are fourteen thousand of them, who know that without their permission they are falsely described as being legislated for by the Central Committee. Let their voices be heard. Let 20,000 trained nurses join the College and defeat their enemies.—Ierne.

